# Knowledge and intake of folic acid to prevent neural tube defects among pregnant women in urban China: a cross-sectional study

**DOI:** 10.1186/s12884-021-03893-4

**Published:** 2021-06-21

**Authors:** Mingming Cui, Xiao-Lin Lu, Yan-Yu Lyu, Fang Wang, Xiao-Lu Xie, Xi-Yue Cheng, Ting Zhang

**Affiliations:** Beijing Municipal Key Laboratory of Child Development and Nutriomics, Capital Institute of Pediatrics, #2 YaBao Road, Beijing, 100020 China

**Keywords:** Folic acid, Knowledge, Intake, Pregnancy, Neural tube defects

## Abstract

**Background:**

The prevalence of neural tube defects (NTDs) in China declined during 2000–2017 with periconceptional folic acid (FA) supplementation, which is effective in reducing the risk of birth defects. We aimed to assess the knowledge and actual use of FA among Chinese pregnant women and to explore factors associated with FA use before pregnancy.

**Methods:**

All data were collected in face-to-face interviews during health visits among pregnant women. We collected information about knowledge and use of FA supplements and demographic, socioeconomic, and health status. One maternity and childcare hospital was chosen in each of four cities: Beijing, Huaibei, Kunming, and Haikou. In total, 435 pregnant women were randomly recruited for interviews conducted from June to December 2016.

**Results:**

A total of 428 pregnant women were included in this survey. Of these, 82.0% (351/428) knew that FA can prevent NTDs, and 75.9% (325/428) knew the correct time to take FA. Overall, 65.9% (282/428) of women knew both that FA can prevent NTDs and the recommended time to take FA before pregnancy. Approximately 95.1% (407/428) of women reported having ever taken FA, only 46.3% (198/428) had begun to take FA supplementation before conception, and 64.5% (109/169) of women from rural areas failed to take FA before pregnancy. Women living in northern China (odds ratio [OR] = 1.81, 95% confidence interval [CI], 1.18–2.77), those with unplanned pregnancy (OR = 1.99, 95% CI 1.30–3.04), and highly educated women (OR = 2.37, 95% CI 1.45–3.88) were more likely to know about FA. Women who were homemakers (OR = 1.94, 95% CI 1.21–3.11) and had unplanned pregnancy (OR = 6.18, 95% CI 4.01–9.53) were less likely to begin taking FA before pregnancy.

**Conclusions:**

Our survey showed that most pregnant women knew about FA. Although preconception intake of FA can help to reduce NTDs, improving the rate of FA intake before pregnancy is needed in urban areas of China, especially among homemakers and women from rural areas or with unplanned pregnancy. Campaigns are needed to increase awareness about FA and FA use before pregnancy among rural women, homemakers, and those with unplanned pregnancy and lower education levels.

## Background

Folic acid (FA) is an essential vitamin and is often used as a supplement [1]. Folate is required for fetal growth and development. If the mother’s dietary intake of folate is inadequate during pregnancy, neural tube defects (NTDs) may occur [2, 3]. In many countries, periconceptional FA supplementation has been shown to dramatically reduce the risk of pregnancies complicated with NTDs [4–6].

The United States Public Health Service (USPHS) recommends that reproductive-age women who are capable of becoming pregnant should consume 400 µg FA daily from enriched foods and supplements [7, 8]; many other countries have made similar recommendations to prevent NTDs [9–12]. The World Health Organization recommends periconceptional FA supplementation, that is, all women should take a daily supplement of 400 µg FA from the moment they plan to conceive until 12 weeks of gestation [13]. However, despite these recommendations and national campaigns, periconceptional intake of additional FA remains very low in many countries [14]. Studies in Japan have found that the prevalence of inadequate FA use varies from 84.5% to 96.2%, according to geographic location, and the lack of awareness about the importance of periconceptional FA supplementation is a main reason for inadequate FA intake [15].

In 2009, a health policy called “Supplementing Folic Acid to Prevent NTD” (SFAPN) was initiated by the Chinese government. The program provides FA supplements, free of charge, to all women who have a rural registration (as opposed to urban registration) and who plan to become pregnant [16]. Under this policy, rural women of childbearing age can receive a daily supplement of 400 µg FA supplied at village health posts [17, 18]. There are disparities in the consumption of FA in China, with rural pregnant women reporting low daily FA consumption in comparison with their urban counterparts [19–23]. Data from the China Birth Defect Surveillance Center show that the incidence of NTDs in rural areas (21.11/10,000) is higher than that in urban areas (10.08/10,000), suggesting that a lack of awareness about the importance of FA intake in rural areas might play a critical role in the incidence of NTDs [24].

With the development of urbanization, many pregnant women who now live in cities have migrated from rural areas. Women’s awareness about the need for periconceptional FA supplements and compliance with recommendations has not been fully studied in urban areas of China. However, there are considerable differences in the rates of compliance with preconception FA supplement use among pregnant women according to demographic characteristics such as education and income [25]. Identifying related factors affecting the failure of some women to correctly take preconception FA supplements could help to improve certain strategies aimed at increasing FA use to reduce birth defects. Further evidence is needed in some countries to improve compliance with pre-pregnancy FA supplementation among pregnant women. Thus, the aim of the present study was to better understand the relationship between knowledge about FA and use of FA, as well as to evaluate factors associated with the likelihood of FA use among pregnant women in urban China.

## Methods

### Participant enrollment

According to different geographical characteristics, we randomly selected the cities of Beijing, Huaibei in northern China, and Kunming and Haikou in southern China, for the present investigation. One maternity and childcare hospital was chosen in each of the four cities. Local maternal and childcare hospital workers participated in a training workshop to learn standardized interview methods before we began recruiting study participants. Approximately 105 women were sampled from each hospital. From June to December 2016, a total of 435 married Chinese pregnant women aged 18–43 years were interviewed during pregnant women’s health visits. All participants were fully informed of the study protocol and provided their informed consent. A total of 428 questionnaires were valid, with an effective participation rate of 98.4%. Face-to-face questionnaire surveys were administered in each maternity and childcare hospital. There is a risk of recall bias in self-reported information. The exclusion criteria were pregnant women under the age of 18 years or women who were not pregnant, no verbal informed consent provided, and unable to participate in the survey owing to conditions like alcoholism, psychosis, and cognitive or mental disorders, impairment, or diseases. The study was reviewed and approved by the Ethics Committee of the Capital Institute of Pediatrics in Beijing, China (No. SHERLL2019034).

In estimating the true prevalence of FA intake among the general female population, we assumed that this would be approximately similar to the prevalence reported in China, with 51.4% of Chinese women beginning to take FA supplements 3 months before amenorrhea [23]. Allowing for an error of 5% level of significance (type 1 error), a 95% confidence interval, and a loss to follow-up rate less than 15%, a sample size of 420 pregnant women would be needed to achieve our study objectives.

### Study instrument

The questionnaire used in this study was based on an original survey administered in a previous study [26]. On the basis of the questionnaire, the relevant literature, and recommendations from experts, we included additional questions according to our hypothesis and objectives. The questionnaire comprised three parts: sociodemographic characteristics, obstetric characteristics, and knowledge and use of FA among pregnant women. Sociodemographic characteristics included age, education level, occupation, local residence, district, gestational age at the time of the interview, planned pregnancy, gravidity, maternal parity status, participation in the National Free Preconception Health Examination Project (NFPHEP; provides preconception health examinations and preconception counseling services for women planning pregnancy), and adverse pregnancy outcomes (including preterm birth, preeclampsia, low and very low birth weight, miscarriage, and stillbirth) [27].

### Definitions

At the time of enrollment, interviewers administered a structured questionnaire to study participants to obtain demographic data and information about women’s awareness, knowledge, and use of FA supplements. Knowledge about FA was defined as knowing that FA prevents NTDs and knowing the correct time to take FA. Use of FA was defined as a self-report of ever having taken FA supplements or multivitamins containing FA at any point during the 3 months before pregnancy up to the time of the interview. Pregnant women were asked to recall when they began to take FA supplements. FA intake before pregnancy was defined as a self-report of beginning to take FA at 3 months or 1 month before conception.

### Statistical analyses

The data were processed using EpiData Software 13.0 (http://www.epidata.dk/download.php). All statistical analyses were performed with Stata 10.0 software (StataCorp LLC, College Station, TX, USA). Categorical variables are presented as frequency and percentage and continuous variables are expressed as mean ± standard deviation (SD). These associations were tested using the chi-square or the correlation index depending on the type of variable involved. The statistical level of significance was two-tailed *P* < 0.05. The associations between knowledge and intake of FA among pregnant women were tested using the chi-square test and multivariable logistic regression analysis. The multivariable logistic regression model took into account education, occupation, district, planned pregnancy, and gravidity. Relevant outcomes were presented as odds ratios (ORs) with their 95% confidence intervals (95% CIs).

## Results

### Characteristics of study participants

A total of 428 women were recruited in our study; seven (1.6%) were excluded from the analysis because of missing information for certain key variables. The mean age of our study participants was 28.5 (± 4.6) years. The average gestational age at the time of the interviews was 27.8 (± 10.5) weeks. Gestational age at the time of the interview among 64 (14.9%) pregnant women was less than 12 weeks. A total 213 participants (49.8%) were from northern China (Beijing and Huaibei) and 215 (50.2%) from the south (Kunming and Haikou). A total of 169 participants (39.5%) were migrants from rural areas. Maternal parity status was multiparous in 149 (34.8%) and nulliparous in 279 (65.2%) participants. In total, 236 (55.1%) women were primigravida and 192 (44.9%) were multigravida. A total of 234 (54.7%) participants planned their pregnancy; 145 (33.6%) were homemakers and were not employed. Approximately 21% (90/428) of pregnant women had adverse pregnancy outcomes. Only 25.5% (109/428) of all women and 64.5% (109/169) of women from rural areas had participated in the NFPHEP.

### Knowledge about FA and FA supplementation among periconceptional women

Overall, 351 (82.0%) pregnant women reported that they were aware that FA prevents NTDs. Although 325 (76.0%) women knew the correct time to take FA, the prevalence of both knowing that FA prevents NTDs and knowing the appropriate time to take FA among participants was 65.9% (*n* = 282). Approximately 95.1% (407/428) of women reported having ever taken FA, but only 46.3% (198/428) had begun to take FA supplementation before conception. Of the 198 women who reported beginning FA supplementation before conception, 62.1% (123/198) of participants started taking FA 3 months before conception and 37.9% (75/198) took FA 1 month before conception. Exactly 86.0% (350/407) of participants who reported ever having taken FA supplements purchased these at their own expense; the remaining women were provided with FA at no cost as part of routine care. In total, 88.7% (361/407) of participants took 400 μg FA every day, and approximately 11.3% of women (46/407) took other doses of FA every day. Only 50.8% (165/325) of pregnant women who knew the appropriate time of FA intake took it at the correct time. Additionally, each woman could select three major sources of their knowledge about FA. Approximately 58.9% of pregnant women obtained their FA information from their doctor and in counseling with a general practitioner, 50.4% from relatives and friends, 26.2% from the Internet, and the remainder from other sources (Table 1).

**Table 1:** Awareness, knowledge, and use of folic acid (FA) supplements among pregnant women in China

	N(428)	%
**Aware that folic acid prevents NTDs**		
Yes	351	82.0
No	77	18.0
**Knew correct time to take folic acid**		
Yes	325	76.0
No	103	24.0
**Knowledge about folic acid**^**a**^		
Yes	282	65.9
No	146	34.1
**Use of folic acid among women**		
Yes	407	95.1
No	21	4.9
**Began to take folic acid among women**		
Before pregnancy	198	46.3
After pregnancy	209	48.8
Not taking folic acid	21	4.9
**Use of FA among women knew correct time to take FA( *n* = 325)**		
Before pregnancy	165	50.8
After pregnancy	152	46.8
No taking folic acid	8	2.4
**Began taking FA before pregnancy(*n* = 198)**		
Three months before pregnancy	123	62.1
One months before pregnancy	75	37.9
**Source of folic acid (*n* = 407)**		
Purchase	350	86.0
Free of charge	57	14.0
**Daily dose(*n* = 407)**		
400 μg	361	88.7
Others	46	11.3
**Sources of information on folic acid **^**b**^**(*n* = 351)**		
Doctor / General practitioner counselling	207	58.9
Relatives/friends	177	50.4
The internet	92	26.2
Newspaper/magazine/books	51	14.5
TV/Radio/WeChat	19	5.4
School for pregnant women	11	3.1
Else	6	1.7

^a^Knowledge about folic acid: Includes both knowing that folic acid prevents neural tube defects (NTDs) and knowing the correct time to take folic acid

^b^Sources of information on folic acid: Three response options

### Association of FA knowledge and FA supplementation before pregnancy

Pregnant women from rural areas were less likely than their counterparts from urban areas to know about FA and to begin taking FA before pregnancy (55.0% vs. 73.0%, *P* = 0.000; 35.5% vs. 53.3%, *P* = 0.000). Women with a college or university degree were more likely than women with a primary or secondary school education to know about FA and to begin taking FA at the correct time (76.5% vs. 67.0% vs. 51.7%, *P* = 0.000; 53.6% vs. 48.0% vs. 35.9%, *P* = 0.005). Women who were employed were more likely to know about FA and to begin taking FA before pregnancy than women who were homemakers (69.7% vs. 57.0%, *P* = 0.012; 51.3% vs. 42.2%, *P* = 0.001). There was a significant difference in knowledge about FA among pregnant women living in northern versus southern areas (73.2% vs. 58.6%, *P* = 0.001). Women who had a planned pregnancy were more likely to know about FA and to begin taking FA before pregnancy than those who had an unplanned pregnancy (73.5% vs. 56.7%, *P* < 0.01; 65.4% vs. 23.2%, *P* < 0.01). Women who were multigravida were more likely than those who were primigravida to begin taking FA before pregnancy (51.6% vs. 42.0%, *P* = 0.047). However, women who became pregnant for the first time were more likely to know about FA than those who had multiple pregnancies (70.3% vs. 60.4%, *P* = 0.031). There was a significant difference in knowledge about FA between nulliparous and multiparous pregnant women (69.9% vs. 58.4%, *P* = 0.017). We found a significant difference with respect to beginning to take FA before pregnancy among pregnant women who knew that FA prevents NTDs and knew the correct time to take FA, as compared with women who did not have this knowledge (50.7% vs. 37.7%, *P* = 0.010) (Table 2).

**Table 2:** Association of knowledge about FA with FA supplementation before pregnancy among pregnant women

Variable	Survey sample	Knowledge about FA		***P***	FA intake before pregnancy		***P***
	N(428)	N(282) %			N(198) %		
**Age at conception (years)**							
< 25	77	44	57.1	0.194	29		0.078
25–34	301	205	68.1		140		
≥ 35	50	33	66.0		29		
**Residence**							
Local residents	259	189	73.0	0.000**	138	53.3	0.000**
Immigrants from rural	169	93	55.0		60	35.5	
**Gestational age(weeks)**							
≤ 12	63	44	69.8	0.474	33	52.4	0.291
≥ 13	365	238	65.2		165	45.2	
**Education**							
Primary/Secondary	145	75	51.7	0.000**	52	35.9	0.005*
College	100	67	67.0		48	48.0	
University	183	140	76.5		98	53.6	
**Occupational status**							
Employed	300	209	69.7	0.012*	154	51.3	0.001*
homemaker	128	73	57.0		54	42.2	
**District**							
Southern	215	126	58.6	0.001*	100	46.5	0.917
Northern	213	156	73.2		98	46.0	
**Planned pregancy**							
Yes	234	172	73.5	0.000**	153	65.4	0.000**
No	194	110	56.7		45	23.2	
**Gravidity**							
primigravidas	236	166	70.3	0.031*	99	42.0	0.047*
multigravidas	192	116	60.4		99	51.6	
**Maternal Parity status**							
nulliparous	279	195	69.9	0.017*	129	46.2	0.989
multiparous	149	87	58.4		69	46.3	
**Adverse pregnancy outcomes**							
Yes	90	59	65.6	0.940	48	53.3	0.130
No	338	223	66.0		150	44.4	
**Participate in NFPHEP**^**a**^							
Yes	109	69	63.3	0.510	59	54.1	0.056
No	319	213	66.8		139	43.6	
**Knowledge about FA**							
Yes	282	-			143	50.7	0.010*
No	146	-			55	37.7	

^*^P < 0.05, **P < 0.001

^a^NFPHEP: National Free Preconception Health Examination Project

We applied multivariable logistic regression models to identify the effects of the investigated factors on knowing about FA and taking FA supplements before pregnancy. A total of seven variables with a *P*-value ≤ 0.05 (Table 2) in the analysis were entered into the multivariable logistic regression model for knowledge about FA. A total of six variables with *P* ≤ 0.05 (Table 2) were entered into the multivariable logistic regression analysis for taking FA before pregnancy. Table 3 shows that women with a university degree were more likely to know about FA (women with a primary/secondary education formed the reference group for the exposure, adjusted OR = 2.37, 95% CI 1.45–3.88). Women who were primigravida were more likely to know about FA (multigravida women were the reference group for the exposure, adjusted OR = 1.54, 95% CI 1.01–2.36). Additionally, women who had a planned pregnancy were more likely to know about FA and to take FA before pregnancy (women who had unplanned pregnancy were the reference group for the exposure, adjusted OR = 1.99, 95% CI 1.30–3.04; adjusted OR = 6.18, 95% CI 4.81–9.53). Pregnant women in northern China were more likely to know about FA (southern China was the reference group for the exposure, adjusted OR = 1.81, 95% CI 1.18–2.77). Women who were employed were more likely to start taking FA before pregnancy (women who were homemakers formed the reference group for the exposure, adjusted OR = 1.94, 95% CI 1.21–3.11)**.**

**Table 3:** Multivariable analysis of folic acid (FA) knowledge and FA supplementation before pregnancy among pregnant women

	**Adjusted OR**	**95% CI**	***P***
**Knowledge about folic acid**			
Education1(college)	1.60	0.92–2.77	0.093
Education2(university)	2.37	1.45–3.88	0.001*
Gravidity	1.54	1.01–2.36	0.049*
Planned pregancy	1.99	1.30–3.04	0.002*
District	1.81	1.18–2.77	0.006*
**Begin to take folic acid before pregnancy**			
Occupational status	1.94	1.21–3.11	0.006*
Planned pregancy	6.18	4.01–9.53	0.000**

^*^P < 0.05, **P < 0.001

Abbreviations: *OR* odds ratio. *CI* confidence interval

Variables included in the multivariable logistic regression analysis: education 1 (college vs. primary/secondary school), education 2 (university vs. primary/secondary school), gravidity (primigravida vs. multigravida), planned pregnancy (planned vs. unplanned), district (north vs. south), and occupation (employed vs. homemaker)

## Discussion

We assessed the relationship between knowledge about FA and use of FA, as well as factors affecting FA supplementation before pregnancy among pregnant women in four cities (Beijing, Huaibei, Kunming, and Haikou) of China. Approximately 82% of women in this study knew that intake of FA during the periconceptional period is an effective way of preventing NTDs, which is higher than the rates of FA awareness in Israel, Turkey, Canada, Qatar, Egypt and Iran [14, 28–32]. The best method to prevent NTDs is to ensure that women of childbearing age are taking appropriate amounts of FA daily. This requires them to be aware of when and how to take FA supplements. The present study found that most women were aware of the benefits of FA supplementation, but only 65.9% of them knew both that FA prevents NTDs and the appropriate time to take FA. To remind pregnant women about the importance of taking FA at any point during the 3 months before pregnancy, in the future, the content of public health information should be more detailed, emphasizing the correct time and importance of taking FA.

Regarding factors related to knowledge about FA, our study found that in northern and southern China, gravidity (primigravida and multigravida status), educational level, planned pregnancy, and residence were related to the total rate of knowledge about FA. This finding is consistent with other studies in the literature reporting that women with higher education levels are more likely to correctly take FA-containing supplements than those with low education levels [18]. Pregnant women with lower education levels may lack awareness, knowledge, and confidence regarding the national recommendations for FA supplementation. They might be unaware of the benefits of FA for healthy fetal growth and development, and the potential susceptibility to and severity of NTDs caused by folate deficiency [33]. Pregnant women with higher education levels have greater awareness and knowledge about FA, can accept new information and master the correct method of taking FA as soon as possible, and have more ways to obtain knowledge, which makes it easier for these women to access FA-related information and take better care of their own health. Therefore, the awareness about FA among pregnant women with university-level educations was significantly higher than that among pregnant women with low education levels.

We found that women living in the north of China had greater awareness about FA than those living in the south. Since 1993, the Chinese government has developed strategies targeting women of childbearing age to promote the use of FA for the prevention of NTDs [18]. Previous studies have also investigated FA intervention programs carried out in the north prior to the national program and implementation of health education because the prevalence of NTDs in the north is higher than that in the south [26]. We found that women who were multigravida were less likely to know about FA than those who were primigravida, but this relationship was not statistically significant. Women who were multigravida were more likely to take FA before pregnancy than those who were primigravida. This difference between “knowing” and “doing” may be owing to a recall bias among pregnant women regarding details before pregnancy. In fact, women who are multigravida are more likely to receive counseling services and information about FA and its role in preventing NTD, resulting in better compliance with FA intake. This finding is also consistent with a study among Lebanese women [34]. Concerned public health experts should continue to call for the reimbursement of FA supplements. Physicians dealing with women of childbearing age should be reminded to recommend FA during prenuptial visits [34]. A few previous studies have reported that the rates of knowledge about FA among women who have migrated from rural to urban areas were not high; this issue requires greater attention. Most pregnant women who have migrated from rural areas have low educational levels. Our study also showed that pregnant women with unplanned pregnancy were less likely to know about the importance of FA during pregnancy. Our study indicated that only half of the pregnant women who knew the correct time of FA intake took it at the right time. We also found that pregnant women who had planned pregnancy had a greater likelihood of knowing about FA (adjusted OR = 1.99, 95% CI 1.30–3.04), which was consistent with other studies [34]. This finding can be explained by the fact that Chinese women who have unplanned pregnancy and who migrate from rural areas are less likely to receive counseling services and information about taking FA at the right time. Strategies to increase awareness about FA supplementation to prevent NTDs among these women are important.

The Chinese government launched a campaign targeting rural areas to increase awareness about the role of FA in preventing NTDs [17]. FA supplementation measures implemented by the government of China to decrease the prevalence of NTDs and other birth defects include NFPHEP preconception counseling services to provide education about the importance of FA supplementation and increase awareness about FA and SFAPN, thereby improving levels of knowledge and FA intake. In this study, more than one-third (60/169) of pregnant women who migrated from rural areas did not participate in the NFPHEP, including some women with unplanned pregnancies or who did not know about the project. Additionally, physicians such as those responsible for preconception health assessments, obstetricians and gynecologists, and general practitioners are the main sources of information about FA. Among women who reported that they had ever heard of FA, 58.9% stated that they had received this information from one of the above medical professionals. Consistent with previous studies conducted in the Mediterranean region and in Glasgow [35], our study suggested the need for ongoing education among health care providers who are most likely to interact with women during the reproductive period before conception. Further action should be taken by policy makers to reinforce supervision of the quality of the health care network to ensure that women obtain sufficient knowledge about FA. At present, most people pay more attention to the safe use of drugs and health products, but the lack of professional knowledge and standardized access to information regarding intake of FA is not optimal. The target population cannot be covered completely only via the information provided by doctors. Therefore, population characteristics and needs must be identified to target and enrich FA knowledge channels such as webblog, WeChat, and text messaging (SMS) [18]. This can improve access to FA knowledge and increase awareness about FA in the target population.

Neural tube closure occurs between days 22 and 28 after conception, and therefore increased FA intake before pregnancy is strongly advised, but this is often not achieved [36]. Our findings indicated that 95.1% of women took FA supplements before and after pregnancy and fewer than half of them began to take FA before becoming pregnant. This was higher than the proportions reported in previous studies conducted in Canada and northern China [26, 29]. Additionally, only 35.5% of women from rural areas began taking FA before pregnancy. Consistent with previous studies [29], only 28.7% (123/428) of our participants had begun to take FA supplementation 3 months before conception and 17.5% (75/428) started taking FA 1 month before conception. Our study indicated that only half of the pregnant women who knew the correct time of FA intake took it at the right time. Women who knew about FA were significantly more likely to take FA before pregnancy than those who did not (*P* = 0.010). Consistent with previous studies [18, 37], poor awareness and knowledge regarding FA is a risk factor that contributes to this poor compliance with taking FA at the right time. Therefore, the target population should be made fully aware of the importance of taking FA during pregnancy via various forms of health education, so that they can master the correct time and dose of taking FA, thereby increasing the rate of FA intake.

Regarding factors related to taking FA at the appropriate time, our findings showed that factors that contribute to poor compliance with taking FA supplements before pregnancy may include unplanned pregnancy and being a homemaker. Consistent with another study [38], our findings showed that pregnant women who were employed were more likely to take FA at the correct time (adjusted OR = 1.94, 95% CI 1.21–3.11) than women who were not employed or who were homemakers. Pregnant women with an occupation have their own economic resources, and their financial independence is stronger than women who are not working or who are homemakers. The rate of taking FA among pregnant women with an occupation was significantly higher than that among unemployed women or homemakers. The health level of individuals is related to socioeconomic conditions. Most pregnant women who purchase FA supplements or compound vitamins containing FA for themselves have sufficient financial resources through their own employment. Consistent with a previous study [39], we found that pregnant women who had a planned pregnancy had a higher likelihood of taking FA at the appropriate time (adjusted OR = 6.18, 95% CI 4.01–9.53). Our findings showed that pregnancy planning was positively associated with the consumption of FA supplements during the periconception period. This could be explained by the fact that women did not take FA until they became pregnant, and compliance among pregnant women with taking FA is poor. Despite recommendations and national campaigns, intake of additional FA at the correct time remains insufficiently high in some cities of China. This finding can be explained by the fact that Chinese women who have unplanned pregnancy and who migrate from rural areas are less likely to receive counseling services and information about taking FA at the right time and may therefore miss the best time to take FA, whereas women who have had a healthy live birth may believe that there is no need to take FA because they have given birth successfully. In the SFAPN and NFPHEP programs, doctors oversee nutritional education, providing FA supplements and following up on the use of FA during the periconceptional period. Improving pregnancy planning (including preconception health examinations and preconception counseling services) could improve awareness and intake of FA during the periconceptional period. Women who are planning pregnancy seek maternal and gynecological care from doctors or general practitioners, including attending regular monthly or bi-monthly follow-up visits to record the condition of their pregnancy and improve management. It is easy to follow up and monitor FA use in the daily activities of these women because they have greater opportunities to consult with doctors or general practitioners and receive more information about FA. To give birth to a healthy baby and prevent birth defects, women planning pregnancy should pay greater attention to their health before as well as during pregnancy. Pregnancy planning is an ideal time to adopt behaviors that can improve the health of both mother and fetus [29]. It is important to encourage pregnancy planning among women, especially those who have migrated from rural to urban areas to improve the rate of FA intake before pregnancy.

### Strengths and limitations

Our study has several strengths. To our knowledge, this is one of the few studies to report results on the prevalence of FA use among pregnant women in urban China, especially women from rural. We evaluated whether this was effective in increasing knowledge about and compliance with FA supplement use among pregnant women. Additionally, we provided evidence for further effective nutrition strategies on promoting FA supplementation intake at the correct time to reduce the burden of NTDs in urban areas of China.

Our study also has several limitations. There is a risk of recall bias as information about FA awareness and use relevant to the risk of NTDs was self-reported. However, we think that the likelihood of recall bias regarding FA use was minimal as the study was conducted at a time during pregnancy when women were taking FA supplements to prevent NTDs. Additionally, our study was conducted in only four cities; therefore, our findings cannot be generalized to other areas. Lastly, this study was a cross-sectional survey; thus the observed relationships cannot establish causality.

## Conclusions

Despite the fact that most of our respondents knew about FA, the actual rate of FA use at the appropriate time during the pre-pregnancy period was not high among our population of women in urban China. Women living in southern China, those who had unplanned pregnancy, women with lower education levels, and those who were immigrants from rural areas had lower levels of knowledge about FA. Women who were homemakers, had migrated from rural regions, and those who had an unplanned pregnancy were less likely to begin taking FA during the pre-pregnancy period. These findings suggest that public health campaigns are urgently needed, with the aim to increase awareness, knowledge, and use of FA at the correct time among women of reproductive age. Special attention should be given to less-educated women, homemakers, those living in southern China, women with unplanned pregnancy, and those who have migrated from rural areas. It is also necessary to encourage pregnancy planning to improve the rate of FA intake among women before pregnancy.

## Data Availability

The manuscript is original. Data and materials are available. The datasets used and analyzed during the current study are available from the corresponding author on reasonable request.

## Abbreviations

NTDs: Neural tube defects
FA: Folic acid
USPHS: The United States Public Health Service
WHO: World health organization
SFAPN: Supplementing Folic Acid to Prevent NTD
NFPHEP: National Free Preconception Health Examination Project
SD: Standard deviation
OR: Odds ratio
CI: Confidence interval
